# The Incidence and Outcomes of Recurrence of Infection after Therapeutic Penetrating Keratoplasty for Medically-Uncontrolled Infectious Keratitis

**DOI:** 10.3390/jcm9113696

**Published:** 2020-11-18

**Authors:** Jayoon Moon, Chang Ho Yoon, Mee Kum Kim, Joo Youn Oh

**Affiliations:** 1 Department of Ophthalmology, Seoul National University College of Medicine, 103 Daehak-ro, Jongno-gu, Seoul 03080, Korea; ja-yoon88@hanmail.net (J.M.); ifree7@gmail.com (C.H.Y.); kmk9@snu.ac.kr (M.K.K.); 2 Laboratory of Ocular Regenerative Medicine and Immunology, Biomedical Research Institute, Seoul National University Hospital, 101 Daehak-ro, Jongno-gu, Seoul 03080, Korea

**Keywords:** cornea, infection, infectious keratitis, recurrence, therapeutic penetrating keratoplasty

## Abstract

Background: This study aimed to investigate the outcome of therapeutic penetrating keratoplasty (TPK) for medically-uncontrolled infectious keratitis, and to determine the factors associated with the recurrence of infection after TPK. Methods: A 10-year retrospective study of medically-uncontrolled infectious keratitis with positive culture results, who received TPK at a tertiary referral center in Korea was performed. Data collection included patient demographics, medical history, pre- and post-operative findings, surgical procedures, causative microorganisms, and visual acuities (VA). The primary outcome measure was the recurrence of infection after TPK, and the factors were compared between patients with and without recurrence. Results: A total of 19 patients (19 eyes) were analyzed, of which 6 eyes (31.6%) had infection recurrence at 21.6 ± 22.84 months after TPK. Recurrence occurred more frequently in the female sex (vs. male, *p* = 0.013) and in longer duration (>30 days) from infection onset to TPK (vs. ≤30 days, *p* = 0.025). Final best-corrected-VA was poorer in patients with recurrence than those without (LogMAR 1.60 ± 0.97 vs. 2.40 ± 0.46, *p* = 0.026). Evisceration was performed in 2 out of 6 patients with recurrence (33.3%), while none was performed in those without recurrence (*p* = 0.028). Conclusion: Infection recurrence after TPK was 31.6%. Given the poor outcome of TPK in eyes with recurrence, close monitoring and intensive treatment are required post-TPK.

## 1. Introduction

The incidence of infectious keratitis is increasing in developed countries, with the rise of contact lens use, corneal surgery, or ocular surface disorders, including dry eye disease [1,2,3]. Various microorganisms, including bacteria, fungus, virus, or *Acanthamoeba* [4,5,6], can cause infectious keratitis, and the causative organisms largely vary by region. In a recent Asian multicenter study, the *Fusarium* species (18.3%) was reported to be the most common microorganism isolated from infectious keratitis, followed by *Pseudomonas aeruginosa* (10.7%) [3], but more than half of the cases analyzed in the study were from India. In our own series involving Korean patients, coagulase-negative *Staphylococci* (15.9%) was the most frequently isolated microorganism, followed by *Staphylococcus aureus* (12.1%) and *Pseudomonas aeruginosa* (10.3%) [6].

In recent years, early and accurate diagnosis became more possible with the introduction of new technologies such as in vivo confocal microscopy or next-generation sequencing [4]. Moreover, a majority of infectious keratitis is still responsive to currently-available topical antibiotics or antifungal agents, although several concerns regarding region-specific epidemiology of pathogens and antimicrobial resistance patterns exist [4,6,7,8,9]. Recent studies showed the efficacy of collagen cross-linking as an adjunctive therapy to antimicrobial treatment for infectious keratitis [10]. Despite these significant advances in diagnostic and therapeutic modalities, there are still cases of infectious keratitis that do not respond to maximal medical therapy and instead progress to corneal perforation or endophthalmitis. In these refractive cases, therapeutic penetrating keratoplasty (TPK) was used and was reported to eliminate infection in 69–100% of infectious keratitis [11]. It is, however, well-known that corneal transplantation during active infection was associated with a high graft failure rate of up to 46–60.7% [3,12]. Hence, meticulous assessment of the effects and risks of TPK is important for effective and safe management of infectious keratitis.

In this study, we investigated the outcome of TPK, which was performed in Korean patients with infectious keratitis that was unresponsive to maximal fortified antimicrobial combinations from the following perspectives—(1) eradication of infection (or recurrence), (2) maintenance of globe integrity (or evisceration), (3) achievement of graft clarity, and (4) visual rehabilitation. In particular, we used recurrence of infection as the primary outcome measure of TPK, because previous studies identified recurrence as the most important cause for graft failure [12,13,14,15,16]. Additionally, we analyzed the factors related to the recurrence.

## 2. Materials and Methods

This study was approved by the Institutional Review Board of Seoul National University Hospital (IRB No. 1802-122-924, Seoul, Republic of Korea) and was conducted with adherence to Declaration of Helsinki. The informed consent from patients was waived by the IRB because the study was based on the retrospective review of old charts. This was a retrospective case-series study of patients with positive culture results who received TPK between 2008 and 2017 at Seoul National University Hospital (a tertiary referral center) in Republic of Korea, for infectious keratitis, which was unresponsive to the maximal antimicrobial therapy. From the medical chart review, the following data were collected—(1) demographic information, (2) general medical and ocular history, (3) clinical characteristics from ocular examinations including BCVA and slit lamp biomicroscopic findings, (4) details of surgical procedures, and (5) causative microorganisms obtained from corneal scraping and culture. Excluded from analysis were patients under 18 years of age and with endophthalmitis at disease presentation.

In all patients, microbial cultures from corneal scrapings were performed at initial presentation. Simultaneously, empirical therapy was started with hourly administration of topical broad-spectrum antibiotics, using combinations of fortified antibiotic eyedrops (2.5% vancomycin + 5% ceftazidime) or 0.5% moxifloxacin (Vigamox^®^, Alcon, Fort Worth, TX, USA). In cases where fungal infection was clinically suspected, topical application of antifungal agent (1% voriconazole and/or 0.15% amphotericin) was combined. After initial hourly administration of topical medications, the administration frequency was adjusted according to the clinical response. Types of topical medications were also changed according to the results from microbial identification and antimicrobial susceptibility tests.

TPK was performed when the infection did not respond to the above medical treatment, as determined by progressive corneal ulcer (impending corneal perforation or perforation already present) and increased in infiltration or hypopyon, despite vigorous antimicrobial use. In all patients, full-thickness corneal transplantation was performed to excise the infected cornea entirely. Removal of crystalline lens or intraocular lens (IOL) was combined if infection was extensive, to involve the lens or IOL. After TPK, medical treatments with antimicrobial and antifungal agents was continued and adjusted, based on the microbiology reports. Topical and systemic corticosteroids, as part of post-TPK medication, were not used until infection was completely resolved.

Statistical analysis was performed using the SPSS software for Windows version 22.0 (SPSS, Inc., Chicago, IL, USA). Levene’s test for equality of variances was performed, and continuous data from the two groups were compared using Student’s *t*-test (for equal variances) or Welch’s *t*-test (for unequal variances). Pearson Chi-square test was used to assess differences in categorical variables between groups. Due to the small number of patients included in this study, multiple comparison correction was not applied. Kaplan–Meier survival analysis of TPK was conducted to estimate the median time from disease onset to TPK, and the log-rank test was used to compare the median time between the groups. A probability value of *p* < 0.05 (two-tailed) was considered to be statistically significant. Data were presented as mean ± standard deviations (mean ± SD).

## 3. Results

### 3.1. Recurrence of Infection after TPK

**Figure 1:**
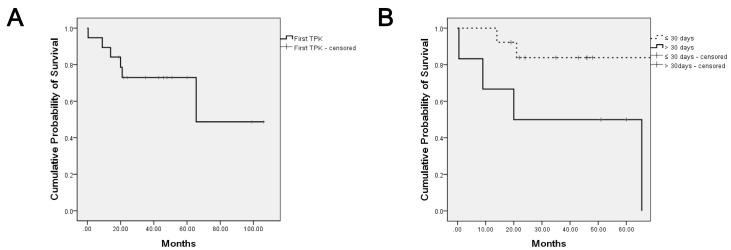
Kaplan–Meier survival curve analysis of first TPK for recurrence. Graph demonstrating Kaplan–Meier survival curves of the first TPK grafts. Survival curves indicate no recurrence of infection. (**A**) Overall infection recurrence time of first TPK is shown. The overall median infection recurrence time was 17 months (mean 21.6 ± 22.84 months, 0.1–65.5). Recurrence-free state at 1 year and 2 years were 89.5% and 73.0%, respectively. (**B**) Dotted-line indicates TPK done within 30 days and solid-line indicates TPK done after 30 days since first symptoms or signs. Median infection recurrence time for TPK done within 30 days and after 30 days were 17.5 months (mean 17.5 ± 4.95, 14–21) and 14.5 months (mean 23.65 ± 29.06, 0.1–65.5), respectively (Log Rank (Mantel-Cox) *p* = 0.030). Recurrence-free state at 1 year were 100% for TPK done within 30 days and 66.7% for after 30 days. Recurrence-free state at 2 years were 83.9% for TPK done within 30 days and 50.0% for after 30 days. TPK = therapeutic penetrating keratoplasty.

In total, 19 eyes of 19 patients underwent TPK at mean 41.3 ± 39.7 days (median 27 days, range 1–150 days) after infection presentation, due to aggravation of infectious keratitis, despite intensive medical treatment (Table 1). There were 11 men and 8 women with an average age of 58.1 ± 19.7 years (range 22–87 years). The right eye was involved in 7 patients (36.8%) and the left eye in 12 (63.2%). Out of 19 patients, 6 patients (6 eyes, 31.6%) had recurrence of infection at the median 17 months (mean 21.6 ± 22.84 months, range 0.1–65.5 months) after an infection-free period, via TPK, during a follow-up of 49.6 ± 29.5 months (range, 11–122 months), while infection did not recur in 13 patients (13 eyes, 68.4%) (Table 1, Figure 1A).

### 3.2. Factors Associated with Recurrence

Demographic, clinical, microbiological, and surgical characteristics were compared between patients, with and without recurrence, and are summarized in Table 1. The factors identified to be significantly associated with the recurrence of infection after TPK, included the female sex (vs. male sex, *p* = 0.013) and longer duration from infection onset to TPK (>30 days vs. ≤30 days, *p* = 0.025). All patients with recurrence (6 of 6 patients) had the presence of crystalline lens at presentation, while 61.5% (8 of 13 patients) of patients without recurrence had crystalline lens; however, this difference was not statistically significant (*p* = 0.077). Additionally, there was no significant difference in the gram stain results between patients with and without recurrence (*p* = 0.248).

In addition, the median time from TPK to infection recurrence was estimated and compared using the Kaplan-Meier survival curves between patients who received TPK within 30 days after infection presentation and those who received TPK after 30 days. The overall median time to recurrence was 17 months, after an infection-free period post-TPK, while recurrence-free state at 1 year and 2 years post-TPK were 89.5% and 73.0%, respectively (Figure 1A). Recurrence-free state at 1 year was 100% for patients that received TPK within 30 days of disease presentation, and 66.7% for those after 30 days. Recurrence-free state at 2 years was 83.9% for patients who had TPK within 30 days of infection presentation and 50.0% for those after 30 days. Further statistical analysis revealed that the median time to recurrence was significantly shorter in eyes receiving TPK, after 30 days of infection than in those receiving TPK within 30 days (14.5 months vs. 17.5 months, *p* = 0.030, Figure 1B).

Other preoperative factors such as systemic immunocompromised state, use of systemic immunosuppressants or topical corticosteroids, presence of hypopyon or corneal perforation, or fungal etiology, were not found to be significantly related to the development of infection recurrence after TPK (Table 1). Likewise, surgical factors including trephination size of donor and recipient corneas, and combined lens extraction at the time of TPK were not significantly associated with recurrence (Table 1).

### 3.3. Outcome of Eyes with Recurrence

Overall, the graft clarity was maintained in 9 of 19 eyes (47.4%) at the final visit (Table 2). Both functional and anatomical outcomes were significantly poorer in eyes with recurrence of infection after TPK (recurrence group) than in those without recurrence (no recurrence group) (Table 2). The final logMAR best corrected visual acuity (BCVA) were 2.40 ± 0.46 in the recurrence group and 1.60 ± 0.97 in the no recurrence group (*p* = 0.026). The corneal graft clarity at final follow-up was achieved in only one of 6 eyes (16.7%) in the recurrence group, whereas 8 of 13 eyes (61.5%) in the no recurrence group maintained clear grafts (*p* = 0.069). Evisceration was eventually performed in 2 of 6 eyes (33.3%) from the recurrence group because the infection was uncontrolled, despite intensive medical therapy and repetitive TPK, whereas no patient from the no recurrence group received evisceration (*p* = 0.028).

## 4. Discussion

Previous studies demonstrated a high therapeutic efficacy of TPK (80.4–93.7%) for management of infectious keratitis [11,13,14,15,17]. Additionally, recurrence rates of infection after TPK reported were low, ranging from 6.34 to 27.5% [12,13,14,15,18]. Compared to previous reports, our study revealed poorer outcome of TPK. In our patients, infection recurred in 31.6% after TPK, and evisceration was eventually performed in 10.5%, due to uncontrolled infection, despite intensive medical therapy and repetitive TPK. This unfavorable outcome of TPK in our population could be derived from ethnic or regional differences in the virulence of causative microorganisms and patterns of antimicrobial resistance. Alternatively, our criteria for selecting patients for TPK might be different from other groups. From our experience, most microorganisms identified from infectious keratitis were sensitive to the combinations of fortified antibiotics (vancomycin and ceftazidime with or without moxifloxacin) used in our clinic [6]. Hence, medical treatment is often favored over surgery in our practice and, therefore, TPK is performed relatively late after infection onset and only in medically-intractable and more severe cases with virulent microorganisms. In fact, during the same 10-year period as this study, a total of 176 patients (176 eyes) had positive corneal culture results for infectious keratitis, of which only a small portion (19 patients, 10.8%) underwent TPK.

It was reported that TPK performed in the late phase of infection was associated with unfavorable outcome in infectious keratitis [15]. Similarly, our study revealed that patients who received TPK after 30 days of the first symptoms had a significantly higher recurrence rate of infection (66.7%), compared to those that received TPK within 30 days after symptom onset (15.4%). It is possible that subjects who received TPK after 30 days of symptom onset were infected with microorganisms that are more slowly-growing, yet aggressive or unresponsive to the conventional medical treatments, compared to those who received the procedure within 30 days. Therefore, sufficient monitoring of medical response and determining the most appropriate time for TPK, alongside an effort to identify the infectious microorganisms, would be crucial steps in managing severe infectious keratitis.

In addition to the time until TPK, our study identified female sex as a significant factor associated with recurrence of infectious keratitis after TPK. There were 5 women (83.3%) and one man (16.7%) in the recurrence group, while there were 3 women (23.1%) and 10 men (76.9%) in no recurrence group. Moreover, two patients who underwent evisceration due to uncontrolled infection in our study were all women. Interestingly, there are studies reporting that the female sex is more prone to infection and that sex hormones might influence immunity against infection [19,20,21]. Hence, female patients might need more medical attention than male patients, when treating severe infectious keratitis. The sexual difference in the susceptibility and severity of infectious keratitis would be an interesting topic for further investigation.

We acknowledge the limitations of our study as follows. The study design is a retrospective case series with a small sample size. The regression analysis was not performed because the variables were not normally distributed. Additionally, due to the retrospective nature of our study, the data collection relied solely on records from medical charts and photos, which might have a possibility of omitting other risk factors such as contact lens use. According to our records, only one patient out of 19 had a history of using bandage contact lens for descemetocele, and this patient did not have recurrence of infection after TPK. Moreover, a possibility of combined *Acanthamoeba* infection could not be fully excluded, because our routine culture protocol did not include *Acanthamoeba* culture. Treatment plans were set towards managing bacterial or fungal infections, unless *Acanthamoeba* infection was clinically suspected.

Nevertheless, our study clearly demonstrates that the recurrence of infection is not uncommon after TPK (31.6% of recurrence rate) and is associated with high graft failure (52.6%) and evisceration rates (10.5%). Given this poor outcome, it would be prudent to closely monitor and intensively treat infection even after TPK in patients with infectious keratitis.

## Figures and Tables

**Table 1:** General Demographics and Characteristics at Time of First TPK.

	Total (19 Patients)	No Recurrence (13 Patients, 68.4%)	With Recurrence (6 Patients, 31.6%)	*p* Value (No Recur vs. with Recur)
**Demographics**				
Age (years)	58.11 ± 19.69 (22–87)	58.46 ± 20.43 (22–80)	57.33 ± 19.81 (34–87)	0.911 *
Sex, Male: Female	11 (27.9%):8 (42.1%)	10 (76.9%):3 (23.1%)	1 (16.7%):5 (83.3%)	0.013 ^§^
Laterality, Right: Left	7 (36.8%):12 (63.2%)	5 (38.5%):8 (61.5%)	2 (33.3%):4 (66.7%)	0.829 ^§^
**Systemic Conditions**				
Diabetes Mellitus	1 (5.3%)	1 (7.7%)	0 (0%)	0.485 ^§^
Stevens-Johnson Syndrome	3 (15.8%)	1 (7.7%)	2 (33.3%)	0.311 ^§^
History of Herpes Simplex Keratitis	2 (10.5%)	2 (15.4%)	0 (0%)	0.310 ^§^
Current Systemic Immunosuppressant Use	3 (15.8%)	3 (23.1%)	0 (0%)	0.200 ^§^
**Ocular Conditions**				
Recent Ocular Trauma	2 (10.5%)	2 (15.4%)	0 (0%)	0.310 ^§^
Current Topical Steroid Use	7 (36.8%)	6 (46.2%)	1 (16.7%)	0.216 ^§^
PKP History	6 ^a^ (31.6%)	5 (38.5%)	1 ^b^ (16.7%)	0.342 ^§^
**Ocular Findings at Presentation**				
BCVA (logMAR)	2.17 ± 0.55 (0.80–3.00)	2.26 ± 0.48 (1.70–3.00)	1.97 ± 0.68 (0.80–2.80)	0.292 *
Hypopyon	9 (47.4%)	6 (46.2%)	3 (50.0%)	0.280 ^§^
Phakia	14 (73.7%)	8 (61.5%)	6 (100%)	0.077 ^§^
Corneal Perforation	9 (47.4%)	6 (46.2%)	3 (50.0%)	0.876 ^§^
**Microbiological Profile**				
Only Bacteria	13 (68.4%)	9 (69.2%)	4 (66.7%)	0.276 ^§^
Gram Stain, positive: negative	11 (57.9%):5 (26.3%)	9 (69.2%):3 (23.1%) ^c^	2 (33.3%):2 (33.3%) ^c^	
Only Fungus	2 (10.5%)	1 (7.7%)	1 (16.7%)	
Bacteria and Fungus Combined	3 (15.8%)	3 (23.1%)	0 (0%)	
Not identifiable	1 (5.3%)	0 (0%)	1 (16.7%)	
**Surgical Methods**				
Time to TPK Since First Symptoms or Signs (days)	41.26 ± 39.74 (1.00–150.00)	25.62 ± 13.43 (6.00–60.00)	75.17 ± 56.88 (1.00–150.00)	0.086 *
Time to TPK ≤ 30 days: > 30 days	13 (68.4%):6 (31.6%)	11 (84.6%):2 (4.3%)	2 (33.3%):4 (66.7%)	0.025 ^§^
Donor Trephine Size (mm)	8.13 ± 0.36 (7.75–9.00)	8.17 ± 0.41 (7.75–8.50)	8.04 ± 0.19 (7.75–8.25)	0.472 *
Recipient Trephine Size (mm)	7.68 ± 0.36 (7.00–8.50)	7.71 ± 0.42 (7.00–8.00)	7.60 ± 0.14 (7.50–7.75)	0.574 *
Combined Lensectomy	4 (21.1%)	4 (30.8%)	0 (0%)	0.126 ^§^

^a^ Reasons for prior PKP were bullous keratopathy, corneal opacity, and neurotrophic keratitis. ^b^ One Patient, who had prior PKP for perforation due to neurotrophic keratitis, had TPK for uncontrolled fungal (Candida) keratitis and had repetitive fungal (Candida) recurrence. ^c^ No recur vs. With Recur, *p* = 0.248, Pearson chi-square test. BCVA = Best corrected visual acuity, TPK = therapeutic keratoplasty, PKP = penetrating keratoplasty. * *t*-test *p* value. ^§^ Pearson chi-square test *p* value.

**Table 2:** Final Outcomes of First TPK.

	Total ^a^ (19 Patients)	No Recurrence (13 Patients, 68.4%)	With Recurrence (6 Patients, 31.6%)	*p* Value (No Recur vs. with Recur)
Visual Acuity (logMAR)	1.85 ± 0.91 (0.00–3.00)	1.60 ± 0.97 (0.00–2.80)	2.40 ± 0.46 (1.70–3.00)	0.026 *
Final graft clarity	9 (47.4%)	8 (61.5%)	1 (16.7%)	0.069 ^§^
Eventual evisceration	2 (10.5%)	0 (0%)	2 ^b^ (33.3%)	0.028 ^§^
Total FU period (months)	49.63 ± 29.48 (11.00–122.00)	49.62 ± 26.43 (19.00–106.00)	49.67 ± 38.12 (11.00–216.00)	0.997 *

^a^ Out of 19 subjects, 6 subjects had already received TPK due to conditions other than uncontrolled infection (bullous keratopathy, corneal opacity, neurotrophic keratitis), and had uncontrolled infectious keratitis thereafter. ^b^ One patient, who was positive for Candida prior to first TPK, had recurred Candida infection and received a second TPK, where the infection recurred 9 months later, which resulted in evisceration. Another patient had received TPK four times due to infection recurrence and became positive for Candida for the first time, just before evisceration. TPK = therapeutic keratoplasty, FU = follow up. * *t*-test *p* value, ^§^ Pearson chi-square test *p* value.
